# Evolução dos desfechos cirúrgicos em metástases vertebrais de tumores sólidos: Análise comparativa ao longo de 2 décadas *

**DOI:** 10.1055/s-0046-1824731

**Published:** 2026-07-28

**Authors:** Fernando Pacheco dos Santos Ferreira da Silva, Pedro Reggiani Anzuatégui, Ana Valéria Rigolino Teixeira, Fernanda Pinto Garcia, Lucas Emanuel Sauer Larocca, Glauco José Pauka Mello

**Affiliations:** 1 Serviço de Ortopedia Oncológica, Hospital Erasto Gaertner, Curitiba, PR, Brasil; 2 Departamento de Cirurgia, Setor de Ciências da Saúde, Universidade Federal do Paraná (UFPR), Curitiba, PR, Brasil

**Keywords:** coluna vertebral/cirurgia, complicações pós-operatórias, metástase neoplásica, mortalidade, sobrevida, mortality, neoplasm metastasis, postoperative complications, spine/surgery, survival

## Abstract

**Objetivo:**

Avaliar a evolução dos desfechos cirúrgicos em pacientes submetidos à cirurgia para metástases vertebrais de tumores sólidos ao longo de mais de duas décadas em um único serviço terciário.

**Métodos:**

Estudo retrospectivo comparativo envolvendo 2 coortes de pacientes submetidos a tratamento cirúrgico para metástases vertebrais: coorte 1 (2002–2015) e coorte 2 (2018–2024). A sobrevida global foi estimada pelo método de Kaplan-Meier e comparada pelo teste log-rank. Complicações maiores pós-operatórias (graus III–IV) foram analisadas por paciente e comparadas entre as coortes.

**Resultados:**

Foram incluídos 301 pacientes (175 na coorte 1 e 126 na coorte 2). A coorte mais recente apresentou aumento significativo da sobrevida global (log-rank *p* < 0,001), com razão de risco para mortalidade de 0,61 (IC95%: 0,48–0,78) em relação à coorte anterior. Observou-se também melhora da sobrevida precoce nos marcos de 30 e 90 dias. A incidência global de complicações foi semelhante entre os grupos, porém houve redução significativa das complicações locais na coorte 2.

**Conclusão:**

Os desfechos cirúrgicos das metástases vertebrais demonstraram melhora ao longo do tempo, especialmente em relação à sobrevida precoce e à redução das complicações locais. Esses achados sugerem que o refinamento da seleção de pacientes, da estratificação prognóstica e do cuidado perioperatório multidisciplinar pode ter contribuído para essa evolução.

## Abstract (en)

**Objective:**

To evaluate the evolution of surgical outcomes in patients undergoing surgery for spinal metastases from solid tumors over more than two decades in a single tertiary referral center.

**Methods:**

The present retrospective comparative study included 2 cohorts of patients who underwent surgical treatment for spinal metastases: cohort 1 (2002–2015) and cohort 2 (2018–2024). Overall survival was estimated using the Kaplan-Meier method and compared with the log-rank test. Major postoperative complications (grades III–IV) were analyzed per patient and compared between cohorts.

**Results:**

A total of 301 patients were included (175 in cohort 1 and 126 in cohort 2). The more recent cohort demonstrated significantly improved overall survival (log-rank *p* < 0.001), with a hazard ratio for mortality of 0.61 (95%CI: 0.48–0.78) compared with the earlier cohort. Early survival at 30 and 90 days also improved in cohort 2. Overall complication rates were similar between groups; however, cohort 2 showed a significant reduction in local complications.

**Conclusion:**

Surgical outcomes for spinal metastases improved over time, particularly in early survival and the reduction of local complications. These findings suggest that refinement in patient selection, prognostic stratification, and multidisciplinary perioperative care may have contributed to this temporal improvement.

## Introdução

As metástases vertebrais são uma manifestação frequente da disseminação de tumores sólidos e representam causa relevante de dor, instabilidade mecânica e déficit neurológico, com impacto direto na autonomia e na qualidade de vida. 1 Diante desse cenário, a indicação de tratamento cirúrgico deve equilibrar potencial benefício funcional com o risco perioperatório, especialmente em pacientes com doença sistêmica avançada e fragilidade clínica. 2

A cirurgia pode proporcionar benefícios clínicos importantes, como descompressão neural, estabilização da coluna e alívio de dor refratária, sobretudo em cenários de instabilidade e compressão medular. 3 Entretanto, trata-se de intervenções potencialmente extensas, associadas a morbidade considerável, particularmente em pacientes frágeis e com expectativa de vida limitada. Nesse contexto, a decisão entre abordagem cirúrgica e modalidades não operatórias –como radioterapia, terapias sistêmicas e cuidados paliativos – exige seleção criteriosa dos candidatos e integração do tratamento em uma estratégia oncológica global. 4 Ainda assim, evidências contemporâneas sugerem que pacientes adequadamente selecionados podem apresentar melhora clinicamente relevante na qualidade de vida relacionada à saúde após a cirurgia para metástases vertebrais , reforçando a importância de uma indicação individualizada. 1 5

Entre os desfechos utilizados na tomada de decisão, a sobrevida precoce após a cirurgia – especialmente nos marcos de 30 e 90 dias – assume papel central. 5 6 Óbitos precoces tendem a indicar desproporção entre agressividade do procedimento e benefício esperado, razão pela qual limiares como 90 dias são frequentemente empregados como referência pragmática para ponderar risco, benefício e proporcionalidade terapêutica. 7 8

Diversos sistemas de estratificação prognóstica foram propostos com o objetivo de auxiliar a tomada de decisão cirúrgica em pacientes com metástases vertebrais. Entre eles, destacam-se escores clássicos baseados predominantemente na biologia tumoral e na extensão da doença, como os de Tomita e Tokuhashi, bem como modelos que incorporam variáveis clínicas adicionais, incluindo estado funcional e comorbidades. No cenário nacional, ferramentas simples voltadas à avaliação de fragilidade também foram descritas, como o modelo de três preditores de Anzuatégui et al., baseado em variáveis clínicas e laboratoriais de fácil obtenção. 9 10 11 Por fim, modelos preditivos baseados em aprendizado de máquina vêm sendo desenvolvidos com desempenho discriminativo potencialmente superior, porém à custa de maior complexidade e menor aplicabilidade imediata à beira do leito. 12 13

Apesar do avanço no uso de modelos e escores, persistem lacunas relevantes na literatura, sobretudo quando se considera a evolução temporal dos resultados cirúrgicos em um mesmo serviço e em um cenário de transição de práticas, tecnologias e recursos assistenciais. Além disso, séries históricas frequentemente apresentam heterogeneidade na indicação, na técnica e no registro de desfechos, especialmente quando há limitações inerentes à coleta retrospectiva.

Diante disso, o presente estudo teve como objetivo avaliar a evolução dos desfechos cirúrgicos em pacientes com metástases vertebrais de tumores sólidos ao longo de mais de duas décadas em um único serviço, com ênfase na sobrevida precoce, na sobrevida global e na ocorrência de complicações, e discutir fatores potencialmente associados a essa evolução.

## Métodos

Trata-se de um estudo retrospectivo, comparativo e longitudinal, conduzido em um único serviço, que avaliou pacientes submetidos a tratamento cirúrgico para metástases vertebrais secundárias a tumores sólidos. Foram analisadas duas coortes distintas: a coorte 1, composta por pacientes tratados entre 2002 e 2015, de natureza retrospectiva, e a coorte 2, composta por pacientes incluídos prospectivamente entre 2018 e 2024. Apesar das diferenças quanto à sua natureza de origem, ambas as coortes foram avaliadas retrospectivamente no presente estudo, com base em dados clínicos e cirúrgicos previamente registrados.

No tocante à técnica cirúrgica, em ambas as coortes o tratamento teve como princípio a descompressão neural associada à estabilização instrumentada para manutenção da estabilidade e controle mecânico. Ao longo do período estudado, houve evolução progressiva dos implantes e instrumentais, com aprimoramento da qualidade dos materiais e da disponibilidade de recursos de fixação e reconstrução, refletindo a modernização do arsenal cirúrgico do serviço, sem alteração dos objetivos fundamentais do procedimento. A caracterização do nível operado e da abordagem cirúrgica foi registrada para fins de análise comparativa entre as coortes.

O objetivo primário foi comparar os desfechos cirúrgicos ao longo de mais de 2 décadas de evolução em um mesmo serviço, com ênfase na sobrevida global, bem como na sobrevida nos marcos temporais de 30, 90 e 365 dias, e na incidência de complicações pós-operatórias. Como objetivos secundários, buscou-se determinar a incidência de complicações locais e sistêmicas, caracterizar sua gravidade e identificar fatores associados às diferenças observadas entre as coortes.

As variáveis categóricas foram descritas em termos de frequências absolutas e percentuais, enquanto os desfechos de sobrevida foram expressos por mediana e respectivos ICs95%. As variáveis categóricas foram comparadas entre as coortes pelo teste do Qui-quadrado ou pelo teste exato de Fisher, conforme as frequências esperadas. A sobrevida global foi estimada pelo método de Kaplan-Meier, definida como o intervalo entre o procedimento cirúrgico e o óbito. Pacientes sem registro de óbito até o último contato, incluindo aqueles com perda de seguimento, foram considerados censurados na data do último registro disponível. A comparação entre as curvas de sobrevida das coortes foi realizada por meio do teste log-rank. Adicionalmente, foi utilizado o modelo de riscos proporcionais de Cox para a estimativa de razões de risco (RRs), com seus respectivos ICs95%.

A análise das complicações pós-operatórias foi conduzida segundo duas abordagens distintas. Para fins do presente estudo, consideraram-se clinicamente relevantes apenas as complicações maiores (graus III–IV), de acordo com a classificação de Rampersaud et al. 14 Inicialmente, realizou-se uma análise por paciente, considerando-se a ocorrência de pelo menos uma complicação local ou sistêmica durante o seguimento. De forma complementar, procedeu-se à análise por evento, permitindo que um mesmo paciente contribuísse com mais de uma complicação, sendo esta abordagem apresentada exclusivamente de forma descritiva. A comparação entre proporções foi realizada utilizando-se o teste do Qui-quadrado, sendo empregado o teste exato de Fisher quando as frequências esperadas foram inferiores a cinco. Quando pertinente, foram estimadas RRs com seus respectivos ICs95%. A análise inferencial foi restrita às variáveis com número suficiente de eventos para aplicação adequada dos testes estatísticos; as demais foram apresentadas de forma descritiva.

Todos os testes estatísticos foram bicaudais, adotando-se nível de significância de *p* < 0,05. As análises estatísticas foram realizadas utilizando o software MedCalc Statistical (MedCalc Software Ltd.), versão 17.6.

O estudo foi aprovado pelo Comitê de Ética em Pesquisa sob o número CAAE: 88280725.1.0000.0098, parecer n° 7.556.572, com concessão de dispensa do Termo de Consentimento Livre e Esclarecido.

## Resultados

**Fig. 1:**
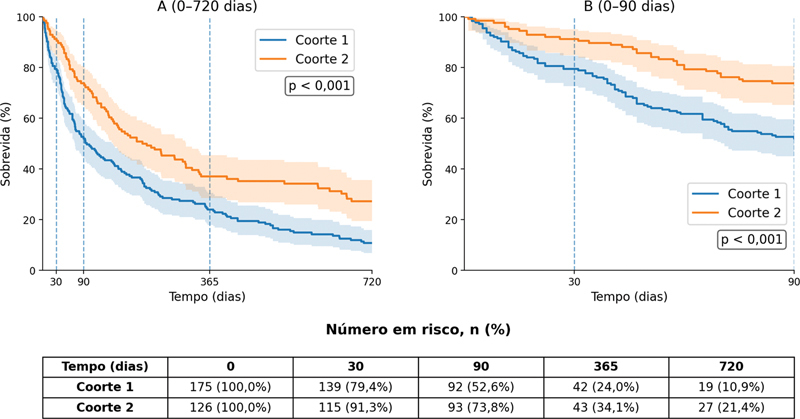
Curvas de Kaplan-Meier da sobrevida global comparando as coortes 1 e 2. ( **A** ) Análise do período de 0 a 720 dias; ( **B** ) ampliação do período inicial (0–90 dias). As áreas sombreadas correspondem aos intervalos de confiança de 95%. A tabela inferior apresenta o número de pacientes em risco em cada marco temporal, expresso em número absoluto e porcentagem (n [%]).

As características demográficas e clínicas das coortes estão apresentadas nas Tabelas 1 2 . Em relação aos desfechos, observou-se diferença na sobrevida global entre as coortes, conforme ilustrado na Fig. 1 . Os parâmetros numéricos da sobrevida global, sua comparação estatística e a síntese nos marcos de 30, 90 e 365 dias estão descritos na Tabela 3 .

**Tabela 1:** Características demográficas da amostra

Variável	Coorte 1, n (%)	Coorte 2, n (%)	Total, n (%)
**Número de pacientes**	175	126	301
**Sexo**			
Masculino	96 (54,9)	58 (46,0)	154 (51,2)
**Idade, anos**			
Média	59,1	58,1	—
Mediana	60,0	58,0	—
**Nível operado**			
Cervical/Cervicotorácico	9 (5,1)	8 (6,3)	17 (5,6)
Torácica	57 (32,6)	53 (42,1)	110 (36,5)
Toracolombar	63 (36,0)	49 (38,9)	112 (37,2)
Lombar/Lombossacro	42 (24,0)	16 (12,7)	58 (19,3)
Múltipla	4 (2,3)	0 (0)	4 (1,3)
**Abordagem cirúrgica**			
Anterior	3 (1,7)	0 (0)	3 (1,0)
Posterior	172 (98,3)	126 (100)	298 (99,0)

**Nota:** Dados expressos em número absoluto (n) e porcentagem (%), quando aplicável.

**Tabela 2:** Tipos histológicos por coorte

Tipo histológico	Coorte 1, n (%)	Coorte 2, n (%)	Total, n (%)	*p*
**Próstata**	51 (29,1)	21 (16,7)	72 (23,9)	0,014
**Mama**	43 (24,6)	37 (29,4)	80 (26,6)	0,358
**Desconhecido**	20 (11,4)	3 (2,4)	23 (7,6)	0,004
**Pulmão**	11 (6,3)	12 (9,5)	23 (7,6)	0,380
**Colo do útero**	12 (6,9)	8 (6,3)	20 (6,6)	1,000
**Cabeça e pescoço**	8 (4,6)	7 (5,6)	15 (5,0)	0,791
**Rim**	7 (4,0)	10 (7,9)	17 (5,6)	0,205
**Trato digestivo**	6 (3,4)	14 (11,1)	20 (6,6)	0,010
**Melanoma**	6 (3,4)	3 (2,4)	9 (3,0)	0,739
**Outro**	6 (3,4)	4 (3,2)	10 (3,3)	1,000
**Sarcoma**	2 (1,1)	4 (3,2)	6 (2,0)	0,241
**Indefinido**	2 (1,1)	2 (1,6)	4 (1,3)	1,000
**Tireoide**	1 (0,6)	1 (0,8)	2 (0,7)	1,000

**Notas** : A categoria “Desconhecido” refere-se a tumores de sítio primário não identificado, caracterizados clinicamente por progressão rápida da doença. A categoria “Outro” inclui, na coorte 1, PNET de membro superior direito (n = 1), tumores de ovário (n = 2), tumor de testículo (n = 1), tumor de adrenal (n = 1) e tumor germinativo (n = 1); e, na coorte 2, tumor paraganglionar (n = 1), tumor de fígado (n = 2) e tumor de vias biliares (n = 1).

**Tabela 3:** Panorama da sobrevida nas coortes

Variável	Coorte 1	Coorte 2	*p*
**Sobrevida ≥ 30 dias, n (%)**	139 (79,4)	115 (91,3)	0,005
**Sobrevida ≥ 90 dias, n (%)**	92 (52,6)	93 (73,8)	< 0,001
**Sobrevida ≥ 365 dias, n (%)**	42 (24,0)	42 (33,3)	0,075
**Sobrevida global: mediana em dias (IC95%)**	97 (71–154)	228 (154–313)	< 0,001
**Razão de risco – coorte 2** ***versus*** **coorte 1 (IC95%)**	—	0,61 (0,47–0,78)	< 0,001

**Nota** : Razão de risco estimada considerando a coorte 1 como referência.

**Fig. 2:**
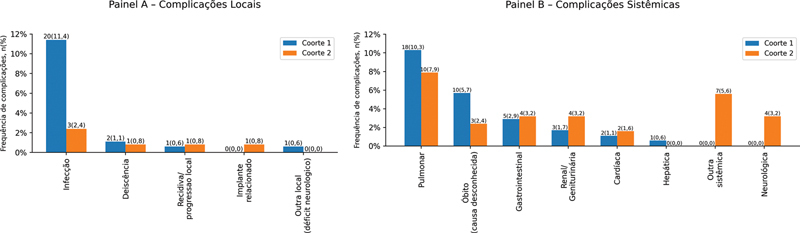
Comparação das complicações locais e sistêmicas entre as coortes. ( **A** ) Complicações locais; ( **B** ) complicações sistêmicas. Valores expressos em número absoluto (N) e porcentagem (%), calculados sobre o total de pacientes de cada coorte. Algumas categorias ocorreram exclusivamente em uma das coortes.

No que se refere às complicações, a incidência global por paciente e por evento encontra-se apresentada na Tabela 4 . A comparação entre as coortes demonstrou diferença estatisticamente significativa apenas para as complicações locais. O detalhamento descritivo das complicações locais e sistêmicas encontra-se ilustrado na Fig. 2 e nas Tabelas 5 6 .

**Tabela 4:** Panorama das complicações nas coortes

Variável	Coorte 1, n (%)	Coorte 2, n (%)	*p*
**A. Pacientes com complicações**			
Qualquer complicação	60 (34,3)	34 (27,0)	0,208
≥ 1 complicação local	23 (13,1)	6 (4,8)	0,017
≥ 1 complicação sistêmica	37 (21,1)	28 (22,2)	0,887
Sem complicações	115 (65,7)	92 (73,0)	0,208
**B. Complicações por evento**			
Eventos de complicações locais	23	6	—
Eventos de complicações sistêmicas	39	34	—
Total de eventos de complicação	62	40	—

**Nota:** O número de pacientes com complicações refere-se a indivíduos que apresentaram ao menos um evento adverso durante o seguimento. O número de complicações por evento corresponde ao total de eventos registrados, podendo ocorrer mais de uma complicação por paciente. Percentuais e cálculo de p não são apresentados para o número de complicações por eventos, pois estes representam frequências absolutas e não constituem observações independentes.

**Tabela 5:** Descrição das complicações locais e sistêmicas da coorte 1

Tipo de complicação	n (%)	Subtipo	n (%)
**Complicações locais**			
Infecção	20 (11,4)	Infecção de ferida operatória	17 (9,7)
		Infecção de ferida operatória + deiscência	2 (1,1)
		Hematoma infectado	1 (0,6)
Deiscência	2 (1,1)	Deiscência de ferida operatória	2 (1,1)
Outra	1 (0,6)	Paralisia no primeiro dia de pós-operatório (déficit neurológico local)	1 (0,6)
**Complicações sistêmicas**			
Pulmonar	18 (10,3)	Infecção pulmonar (pneumonia ± sepse/óbito)	14 (8,0)
		Insuficiência respiratória não infecciosa	3 (1,7)
		Tromboembolismo pulmonar	1 (0,6)
Óbito (causa desconhecida)	10 (5,7)	—	10 (5,7)
Gastrointestinal	5 (2,9)	Hemorragia digestiva alta	3 (1,7)
		Hemorragia digestiva baixa	2 (1,1)
Renal/Geniturinária	3 (1,7)	Insuficiência renal aguda	2 (1,1)
		Infecção do trato geniturinário	1 (0,6)
Cardíaca	2 (1,1)	Insuficiência cardíaca congestiva (com óbito)	1 (0,6)
		Complicação cardíaca isquêmica	1 (0,6)
Hepática	1 (0,6)	Insuficiência hepática aguda	1 (0,6)

**Notas:** Valores expressos em número absoluto (N) e porcentagem (%) calculada sobre o total de pacientes da coorte. “—” indica ausência de subtipo aplicável.

**Tabela 6:** Descrição das complicações locais e sistêmicas da coorte 2

Tipo de complicação	n (%)	Subtipo	n (%)
**Complicações locais**			
Infecção	3 (2,4)	Infecção de ferida operatória	3 (2,4)
Deiscência	1 (0,8)	Deiscência de ferida operatória com nova sutura	1 (0,8)
Recidiva	1 (0,8)	Progressão local tumoral com paraplegia precoce	1 (0,8)
Implante relacionado	1 (0,8)	Soltura de parafuso/reoperação	1 (0,8)
**Complicações sistêmicas**			
Pulmonar	10 (7,9)	Pneumonia	7 (5,6)
		Insuficiência respiratória não infecciosa	2 (1,6)
		Derrame pleural	1 (0,8)
Outra sistêmica	7 (5,6)	Distúrbio metabólico/hematológico sistêmico	3 (2,4)
		Infecção de partes moles	2 (1,6)
		Infecção sistêmica viral (doença do coronavirus 2019)	1 (0,8)
		Sepse sem foco definido	1 (0,8)
Neurológica	4 (3,2)	Convulsões relacionadas ao sistema nervoso central/tumor	3 (2,4)
		Sintomas neurológicos por metástase cerebral	1 (0,8)
Gastrointestinal	4 (3,2)	Abdome agudo obstrutivo	1 (0,8)
		Hemorragia digestiva alta	1 (0,8)
		Hemorragia digestiva baixa	1 (0,8)
		Pancreatite aguda	1 (0,8)
Renal/Geniturinária	4 (3,2)	Infecção do trato geniturinário	3 (2,4)
		Insuficiência renal aguda	1 (0,8)
Óbito (causa desconhecida)	3 (2,4)	—	3 (2,4)
Cardíaca	2 (1,6)	Parada cardiorrespiratória intraoperatória (com óbito)	1 (0,8)
		Endocardite infecciosa	1 (0,8)

**Notas** : Valores expressos em número absoluto (N) e porcentagem (%) calculada sobre o total de pacientes da coorte. “—” indica ausência de subtipo aplicável.

## Discussão

O presente estudo demonstrou melhora significativa dos desfechos cirúrgicos ao longo de mais de 2 décadas em um mesmo serviço, com destaque para o aumento da sobrevida global e, de forma ainda mais relevante, para a melhora da sobrevida precoce nos marcos temporais de 30 e 90 dias, além da redução das complicações locais. Esses achados sugerem que a evolução observada não se limita a avanços técnicos isolados, mas reflete uma transformação mais ampla na abordagem do paciente com metástases vertebrais, envolvendo seleção mais criteriosa, amadurecimento da tomada de decisão cirúrgica e maior integração multidisciplinar do cuidado. 2 3

A sobrevida precoce após o tratamento cirúrgico constitui um desfecho de grande relevância clínica no contexto das metástases vertebrais. Pacientes que evoluem a óbito nos primeiros 30 a 90 dias após a cirurgia, em geral, apresentam benefício clínico limitado com intervenções extensas, o que reforça a importância de uma indicação cirúrgica precisa e individualizada. Estudos prévios demonstram que a mortalidade precoce está fortemente associada a fatores prognósticos sistêmicos, carga tumoral e reserva funcional, devendo ser considerada centralmente na decisão terapêutica. Nesse cenário, a melhora observada nesses marcos temporais entre as coortes sugere avanço substancial na capacidade de identificar, de forma mais acurada, os pacientes com maior probabilidade de benefício real com o tratamento operatório. 4 5 6

A evolução da estratificação prognóstica emerge, assim, como um dos principais fatores associados à melhora da sobrevida precoce observada neste estudo. Tradicionalmente, sistemas de pontuação clássicos representaram marcos fundamentais na tentativa de sistematizar a indicação cirúrgica em pacientes com metástases vertebrais. Com o avanço das terapias sistêmicas e do suporte perioperatório, há evidências de que alguns desses escores possam subestimar a expectativa de vida de subgrupos específicos, o que motivou comparações contemporâneas entre múltiplos modelos e o desenvolvimento de ferramentas mais refinadas, incorporando variáveis clínicas, laboratoriais e oncológicas com melhor desempenho para predição de sobrevida precoce e global. 7 8 9 10 11

Nesse contexto, modelos preditivos contemporâneos e validações prospectivas demonstraram capacidade discriminatória moderada a boa para desfechos clinicamente relevantes, incluindo mortalidade precoce, reforçando a tendência de evolução da tomada de decisão cirúrgica com foco em reduzir intervenções potencialmente fúteis e maximizar o benefício individual. 12 13 15 16 17 Inserido nessa linha, o modelo preditivo proposto por Anzuatégui et al., 11 desenvolvido em população nacional, representa contribuição importante para o cenário brasileiro. Adicionalmente, uma validação externa prospectiva recente desse modelo de fragilidade de 3 preditores demonstrou desempenho aceitável para predição de sobrevida em 90 dias, reforçando sua utilidade como ferramenta simples de apoio à decisão em cenários clínicos diversos. 18

Além da seleção mais adequada dos pacientes, a melhora dos desfechos cirúrgicos observada ao longo do tempo também pode ser atribuída à evolução do cuidado perioperatório e da técnica cirúrgica propriamente dita. A consolidação de abordagens multidisciplinares, com integração entre cirurgiões ortopédicos oncológicos, oncologistas clínicos, radioterapeutas, anestesiologistas, equipes de terapia intensiva e medicina hospitalar, além de profissionais de estomaterapia, permitiu planejamento terapêutico mais estruturado e manejo pós-operatório mais eficiente, com impacto potencial na padronização de condutas e no cuidado de feridas operatórias. Esse modelo de cuidado integrado tem sido amplamente reconhecido como essencial no tratamento contemporâneo das metástases vertebrais, contribuindo para redução de complicações e melhor controle sintomático. 19 20

A redução das complicações locais observada na coorte mais recente reforça esse conceito. Complicações cirúrgicas, especialmente infecção do sítio cirúrgico, representam fator crítico no equilíbrio risco-benefício da cirurgia em pacientes oncológicos, estando associadas a pior prognóstico funcional e sobrevida. A adoção progressiva de medidas preventivas, como protocolos perioperatórios mais rigorosos, otimização do controle clínico e maior padronização técnica, provavelmente exerceu papel relevante nesse desfecho. 14 20

Adicionalmente, o uso de vancomicina tópica intralesional passou a integrar progressivamente a rotina do serviço, nos últimos anos, como medida adjuvante de prevenção de infecção do sítio cirúrgico. Embora os estudos apresentem resultados divergentes quanto à efetividade dessa estratégia, é possível que sua adoção ao longo do tempo tenha contribuído, ao menos em parte, para a menor frequência de infecções observada na coorte mais recente, em conjunto com outras mudanças relacionadas à evolução técnica e ao cuidado perioperatório. 21 22

Outro aspecto relevante deste estudo reside na descrição pormenorizada das complicações associadas à cirurgia de metástases vertebrais. Enquanto grande parte da literatura aborda esses eventos de forma agregada ou se limita a desfechos binários, como a presença ou ausência de complicações maiores, a caracterização detalhada apresentada na Tabela 5 permite uma compreensão mais fiel da morbidade perioperatória nessa população. Estudos prévios já destacaram as limitações inerentes à captura e à classificação de eventos adversos em cirurgia da coluna, especialmente em pacientes oncológicos, nos quais complicações sistêmicas podem refletir tanto o impacto do procedimento cirúrgico quanto a evolução da doença de base e de suas comorbidades. 14 23

As mudanças observadas no perfil histológico ao longo do tempo também merecem consideração. A redução da proporção de casos com origem primária desconhecida sugere melhora nos métodos diagnósticos e maior integração do paciente ao cuidado oncológico global. No presente estudo, observou-se aumento relativo de metástases de origem do trato digestivo e redução de casos de origem prostática, achados que podem refletir tanto alterações epidemiológicas quanto a maior eficácia dos tratamentos sistêmicos e da radioterapia em subgrupos específicos, com impacto na seleção de pacientes encaminhados para cirurgia. 24 25 26 27 28

Do ponto de vista científico, este estudo apresenta caráter inédito no cenário nacional ao oferecer uma análise robusta e comparativa da evolução do tratamento cirúrgico das metástases vertebrais em população brasileira ao longo de mais de 20 anos, em um único serviço. A escassez de séries nacionais de grande porte com esse escopo limita a extrapolação de dados internacionais para a realidade local, tornando os achados aqui apresentados particularmente relevantes para a prática clínica no Brasil.

Apesar de seus pontos fortes, este estudo apresenta limitações que devem ser reconhecidas. A natureza retrospectiva da análise pode introduzir vieses inerentes à coleta de dados e à completude de informações, especialmente nos períodos mais antigos, quando prontuários físicos e mudanças nas equipes assistenciais podem ter impactado a padronização dos registros. Além disso, a ausência de dados funcionais sistematizados e o potencial viés de indicação cirúrgica ao longo do tempo constituem limitações adicionais. Ainda assim, tais fragilidades refletem a prática clínica real e não invalidam as tendências observadas.

Em síntese, os resultados deste estudo indicam que a melhora da sobrevida precoce e a redução das complicações locais ao longo do tempo estão fortemente associadas ao refinamento da seleção dos pacientes, ao amadurecimento da tomada de decisão cirúrgica baseada em prognóstico e à evolução do cuidado multidisciplinar. Esses achados reforçam o papel da cirurgia como parte de uma estratégia terapêutica individualizada e integrada, voltada a maximizar o benefício clínico e minimizar a morbidade em pacientes com metástases vertebrais. A síntese dos principais pontos desta seção está destacada na Tabela 7 .

**Tabela 7:** Síntese interpretativa das diferenças entre as coortes

Domínio	Racional interpretativo
**Sobrevida precoce**	Melhora da sobrevida global e precoce aos 30 e 90 dias na coorte 2.
**Complicações locais**	A coorte mais recente apresentou menor frequência de complicações locais após o tratamento cirúrgico das metástases vertebrais.
**Estratificação prognóstica**	Ênfase na utilização de estratificação prognóstica na indicação cirúrgica para metástases vertebrais no período mais recente.
**Cuidado perioperatório**	Maior estruturação do cuidado perioperatório multidisciplinar com o passar do tempo, com destaque para o suporte clínico no pós-operatório.

## Conclusão

Ao longo de mais de 2 décadas, observou-se melhora progressiva dos desfechos cirúrgicos em pacientes com metástases vertebrais tratados em um único serviço, com destaque para o aumento da sobrevida precoce e a redução das complicações locais. Esses achados reforçam a importância do refinamento da seleção de pacientes e da estratificação prognóstica, em conjunto com a evolução técnica e o cuidado perioperatório multidisciplinar, para otimizar resultados. Assim, a cirurgia deve integrar uma estratégia terapêutica individualizada, indicada de forma criteriosa para pacientes com maior probabilidade de benefício clínico, visando maximizar ganhos e minimizar a morbidade associada ao tratamento.
